# Evaluation of digit ratio (2D:4D) in breast cancer patients

**DOI:** 10.1038/s41598-024-64692-3

**Published:** 2024-06-14

**Authors:** Şafak Yıldırım Dişli, Ali Caner Özdöver, Elif Yüce, Ahmet Kürşad Dişli, Evren Fidan

**Affiliations:** 1 Department of Medical Oncology, Kayseri City Hospital, Kayseri, Turkey; 2 Department of Medical Oncology, Kanuni Research and Education Hospital, Trabzon, Turkey; 3 Department of Medical Oncology, Tekirdağ City Hospital, Tekirdağ, Turkey; 4 Department of Medical Oncology, Erciyes University, Kayseri, Turkey; 5 Department of Medical Oncology, Karadeniz Technical University, Trabzon, Turkey

**Keywords:** Breast cancer, Digit ratio, hormonal exposure, Cancer, Breast cancer, Cancer therapy

## Abstract

Breast cancer is a hormone-dependent cancer. Hormonal exposure begins in the intrauterine period and continues in later years of life. 2D:4D ratio is accepted as an indicator of this exposure. The aim of this study was to investigate whether there is a difference in 2D:4D ratio between pathological subgroups of breast cancer and healthy control group. In this study, 204 participants, 154 breast cancer patients and 50 healthy control volunteers with similar age distribution, were included. Both hands of all participants were scanned using a digital scanner. The second and fourth finger lengths were measured using a digital measuring ruler with an accuracy of 0.05 mm. The 2D:4D ratio was calculated as the length of the second finger divided by the length of the fourth finger. A total of 204 patients (55 triple negative, 52 luminal B, 33 luminal A, 14 HER2-overexpessing and 50 healthy control volunteers) were subjected to finger scanning. There was no statistically significant difference in mean age between the groups. The right hand 2D:4D ratio was significantly lower in the Luminal A group compared to the other groups (*p* < 0.048). Although prenatal hormonal exposure is accepted as a risk factor for breast cancer, no study has evaluated patients in pathological subgroups. The 2D:4D ratio may be associated with breast cancer especially in the luminal A group in which hormone receptors are strongly positive and which has a better prognosis compared to the other groups.

## Introduction

Breast cancer is the most common malignancy in women worldwide and has a very high mortality rate^1^. Breast cancer is a multifactorial disease in which environmental and genetic factors play a role in its etiology^2^.

Breast cancer is a hormone-dependent cancer and there is a positive correlation between prolonged exposure to estrogen and breast cancer incidence^3,4^. Early menarche, late menopause, first pregnancy at an advanced age, never having given birth, hormone replacement therapies and obesity are factors that increase the risk of breast cancer^5,6^. Although gene mutations such as BRCA1, BRCA2, p53 and PTEN constitute a high risk for breast cancer, they are observed in a small proportion of breast cancer cases^2^.

Development of the breast tissue begins in the intrauterine period and continues throughout puberty, pregnancy and lactation. Estrogen increases mitotic activity in breast cells and causes proliferative effect^6^. Long-term and excessive exposure to estrogen is thought to be involved in the development of breast cancer with a cumulative effect on breast gland tissue throughout life^7–10^.

Many studies have shown that high endogenous estrogen and androgen levels and low sex hormone binding globulin (SHBG) levels are associated with postmenopausal and premenopausal breast cancer^11,12^. Exposure to sex hormones may begin in the intrauterine period and may be associated with cancer development later in life. The ratio of second and fourth fingers (2D:4D) can be used as an indirect method to show the effects of androgen exposure in the prenatal period^6,13^. It is accepted that this ratio is formed in the intrauterine period and does not change throughout life and may be an important and early diagnostic marker for future breast cancer development^14,15^.

The activity of androgen and estrogen receptors is higher in the 4th finger than in the 2nd finger. Androgen and estrogen affect chondrocyte proliferation in different patterns. Decreased androgen decreases growth in the 4th finger and leads to a higher 2D:4D ratio^16^. A lower 2D:4D ratio is associated with more testosterone and less estrogen exposure during the intrauterine period, with a lower 2D:4D ratio expected in males^17^.

Numerous studies have attempted to unravel the complex relationships between prenatal hormone exposure and breast cancer risk. Among these, Manning and Bundred^14^ and Hong, Zhan-Bing et al.^28^ reported potential correlations between the 2D:4D digit ratio and breast cancer risk, suggesting that prenatal hormonal environments may influence susceptibility to this disease. In their prospective cohort study, Muller et al.^10^ provided evidence that the 2D:4D ratio, a marker of prenatal hormone exposure, was associated with factors linked to breast cancer risk, further supporting the idea that prenatal hormonal exposure plays an important role in the etiology of the disease. Similarly, Manning and Leinster^8^ investigated the association between the 2D:4D ratio and the age at onset of breast cancer and suggested that prenatal estrogen exposure may influence the timing of cancer onset, as indicated by the ratio.

Studies have shown that not only breast cancer but also other malignancies may be associated with hormonal exposure in the prenatal period. A meta-analysis on the association of 2D:4D ratio with cancer showed that intrauterine sex hormone exposure may be associated with cancer risk later in life. In this meta-analysis, brain cancer, prostate cancer and gastric cancer were associated with low 2D:4D, while breast cancer and cervical intraepithelial neoplasia were associated with high 2D:4D^18^. In a systematic review published in 2022, of the 25 studies evaluated, high 2D:4D ratio was reported as a possible predictor of cancer risk in 11 studies and low 2D:4D ratio was reported as a possible predictor of cancer risk in 8 studies, while no association between this ratio and cancer risk was shown in 5 studies^19^.

In a study investigating the relationship between 2D:4D ratio and lung cancer, it was reported that low 2D:4D, especially in the right hand, may be associated with the risk of developing lung cancer^20^.

This ratio has been investigated many times for diseases other than cancer. For example, the relationship between thyroid diseases and prenatal steroid hormone exposure has been investigated and the 2D:4D ratio for the left hand was found to be higher in women with thyroid disease^21^. Since it is known that testosterone is associated with muscle mass growth and estrogen affects adipogenesis, the 2D:4D ratio was evaluated to see if it could be an indicator of fat mass, muscle mass and body proportions in children and it was shown that it could be an indicator for muscle mass development in girls in early childhood^22^. In other studies on children, the relationship between 2D:4D ratio and cortisol, vitamin D level and body composition, and the relationship between 2D:4D ratio and fitness level were evaluated^23,24^. There are studies suggesting that prenatal sex steroid exposure may be a risk factor for migraine in adults and may be associated with birth weight, BMI and muscle strength in children^25,26^.

In breast cancer, which mostly develops as a hormone-dependent cancer, prenatal exposure to sex steroids may have an effect on the risk of breast cancer development in the future. Different hormonal conditions are observed in pathologic subgroups of breast cancer. Our study aims to provide new insights into how prenatal hormone exposure may differentially affect breast cancer subtypes by examining the 2D:4D ratio in pathologic subgroups of breast cancer.

## Material and method

This study was conducted between January 2020 and December 2020 with outpatients diagnosed with breast cancer over the age of 18 who were admitted to the oncology outpatient clinic for control and treatment. A total of 159 female patients and 50 healthy female volunteers were included in the study. Five patients who could not fully place their hand on the scanning device due to finger deformity or rheumatic disease were excluded from the study.

The study was planned to include all male and female breast cancer patients, but since there were no male breast cancer patients during the study period, the study was conducted only with female breast cancer patients and the control group was selected from female volunteers.

The control group consisted of 50 healthy volunteers age-matched with breast cancer patients to minimize age-related confounding. Controls were selected from hospital staff and relatives of patients without a history of breast cancer or hormone-related malignancy. Age matching was achieved by selecting control participants within ± 2 years of the mean age of the breast cancer group. Each patient included in the control group underwent a brief screening to verify their health status and history of breast cancer, ensuring the reliability of the comparison between the groups.

Patients who were informed about the study, accepted the study and signed the informed consent form were measured. Karadeniz Technic University Ethics Committees approved this study (Trial No. 24237859-228), which was conducted according to ICH-Good Clinical Practice rules and the Declaration of Helsinki.

**Figure 1:**
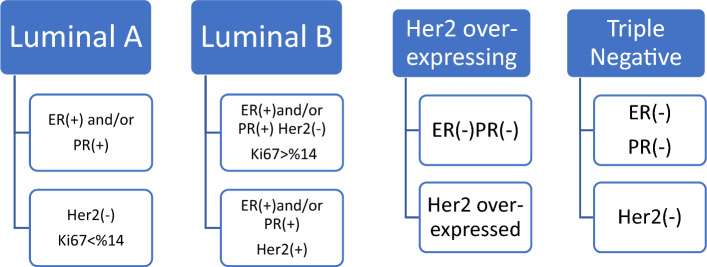
The flowchart of the pathologic subtypes of breast cancer.

Patients were divided into 4 groups as luminal A, luminal B, HER2 over-expressing and triple negative group according to the expression of estrogen receptor (ER), progesterone receptor (PR) and epidermal growth factor receptor (HER2) immunohistochemically according to St Gallen Consensus.ER positive PR positive HER-2 negative patients with low Ki-67 index (< 14) were considered as luminal A; ER and/or PR positive HER-2 negative, Ki-67 index high and ER and/or PR positive HER2 positive patients as luminal B; ER and PR negative HER2 over-expressed patients as HER-2 over-expression; ER PR and HER2 negative patients as triple negative group^27^. Figure 1 demonstrates the flowchart of the pathologic subtypes of breast cancer.

### Digit measurements

The hand of the patients was placed on a fixed point of the digital scanner and the scanning process was performed twice on both hands. From the digital images of the scans, a digital ruler with a precision of 0.05 mm was used by an observer who was unaware of the disease group and measurements were made on the palm of the hand from the apex of the fingers to the metacarpophalangeal flexion crease point, passing through the middle point of the finger. In individuals with more than one wrinkle, the most proximal wrinkle was taken as the basis. After scanning the fingers of the hand twice, the measurements of the fingers of both hands were averaged. Then 2D:4D ratio was obtained by dividing 2D length by 4D length. The difference between right and left 2D:4D was calculated as Δr − l.

### Statistical analysis

Statistical analyses were performed using "IBM SPSS Statistics for Windows. Version 25.0 (Statistical PackagefortheSocialSciences, IBM Corp., Armonk, NY, USA)". Descriptive statistics are presented as n and % for categorical variables and Mean ± SD for continuous variables.

When the data of the study were analysed in terms of normality assumptions, Kolmogorov–Smirnov values were determined as *p* > 0.05. Therefore, ANOVA test, one of the parametric tests, was applied to determine whether there was a significant difference between the groups with Age, Right 2D:4D, Left 2D:4D and Difference 2D:4D values. In case of a significant difference between the groups, Bonferroni post-Hoc test was performed to determine between which groups the significance was between. *P* < 0.05 was considered statistically significant.

### Ethical approval

The present study was approved by the Karadeniz Technic University Ethics Committees and was conducted in compliance with ICH-GCP rules and Declaration of Helsinki. Trial No. 24237.

### Informed consent

Only patients with documented willingness to the use of their medical data for research were included.

## Results

A total of 204 patients were included in the study. Among the patients, 55 (27%) were TN, 52 (25.5%) Luminal B, 33 (16.2%) Luminal A, 14 (6.9%) HER-2 over-expressing patients and 50 (24.5%) were control group (Table 1).

**Table 1:** Demographic characteristics of the patients participating in the study.

Variant	
Study groups, n(%)	
Triple Negative	55(%27)
Luminal B	52(%25.5)
Luminal A	33(%16.2)
HER2overexpressing	14(%6.9)
Control	50(%24.5)
Chronic disease, n(%)	
Hypertension	25 (%16.2)
Diabetes mellitus	16 (% 10.3)
Thyroid diseases	14 (%9)
Other	23 (%14.9)
N/A	76 (%49.3)
Stage, n(%)	
Stage I	33 (%21.4)
Stage II	55 (%35.7)
Stage III	44 (%28.6)
Stage IV	22 (%14.3)
Treatment status, n(%)	
Follow-up without treatment	33 (%21.4)
Receiving treatment	121 (%78.6)
BMI, mean	
Luminal A	29
Luminal B	31.6
Triple Negative	27.1
Her2 overexpressing	34
Control	27.7

The mean age of the patients who participated in the study was 51.39 ± 12.59 years. As seen in Table 2, there was no statistically significant difference between the groups in terms of mean age (*p* = 0.126).

**Table2:** Comparison of mean age by groups.

Average age, mean ± SD	51.39 ± 12.59
Groups age, mean ± SD	
Triple Negative	48.85 ± 14.09
LuminalB	51.33 ± 10.32
LuminalA	55.45 ± 13.01
HER2over-expressing	52 ± 11.48
Control group	50.30 ± 10.61
	p = 0.126

ANOVA test, Post-Hoc: Bonferronitest, *p* < 0.05 is statistically significant.

**Table 3:** Comparison of 2D:4D ratios of the patients with breast cancer and control subjects.

	^1^Triple Negative (n = 55)	^2^Luminal B (n = 52)	^3^Luminal A (n = 33)	^4^Her2Enrich (n = 14)	^5^Control (n = 50)	p	Post-hoc
Right 2D:4D, Mean ± SD	0.9439 ± 0.04	0.9389 ± 0.04	0.9096 ± 0.04	0.9649 ± 0.04	0.9553 ± 0.03	**0.048**	3 < 1, 4, 5
Left 2D:4D, Mean ± SD	0.9808 ± 0.03	0.9751 ± 0.14	0.9751 ± 0.06	0.9894 ± 0.03	0.9700 ± 0.04	0.571	–
Δr − l 2D:4D, Mean ± SD	− 0.0249 ± 0.05	− 0.0319 ± 0.06	− 0.0281 ± 0.04	− 0.0136 ± 0.04	− 0.0238 ± 0.04	0.229	–

No statistically significant difference was found between the groups in terms of left hand and right-left hand ratio difference (*p* = 0.571, *p* = 0.229, respectively).

A statistically significant difference was found between the groups in terms of right hand (*p* = 0.048). Right hand ratio was significantly higher in TN group (*p* = 0.035, Bonferroni test), Her2+ group (*p* = 0.019, Bonferroni test) and control group (*p* = 0.006, Bonferroni test) compared to Luminal A patients (Table 3).

Significant values are in [bold].

## Discussion

There are limited studies in the literature investigating the association of intrauterine sex hormone exposure with breast cancer^10,28^. High intrauterine exposure to sex hormones, especially maternal estrogen and umbilical cord dehydroepiandrosterone concentrations have been found to increase breast cancer^29,30^. The number of studies investigating the 2D:4D ratio, which is considered as an indirect indicator reflecting exposure to sex hormones, in breast cancer is limited compared to other cancers. One of these studies is a prospective cohort study published in 2012. In this study, it was reported that the 2D:4D ratio may be associated with breast cancer risk and age at breast cancer onset^10^. In 2014, another study conducted in Chinese women with breast cancer investigated the 2D:4D ratio and found that this ratio was significantly higher in patients with breast cancer^28^. There are no studies comparing the 2D:4D ratio with pathological subtypes of breast cancer.

In our study, we immunohistochemically divided breast cancer patients into 4 subgroups. No statistically significant difference was observed between the groups in terms of age at diagnosis. We found a lower 2D:4D ratio in the right hand in estrogen and/or progesterone receptor positive HER2 negative luminal A patients, which constitute approximately 60% of breast cancer and have a better prognosis than other subtypes^31^. Although hormone receptor positivity was also found in the Luminal B patient group, no such association was found in Luminal B and other subtypes. This observation suggests a complex relationship between prenatal hormone exposure and the risk of developing certain subtypes of breast cancer. The Luminal A subtype, known for its hormone receptor positivity, low Ki-67 proliferative index and better prognosis, suggests that it presents a distinct profile that may be more directly affected by prenatal hormonal environments compared to other breast cancer subtypes. The absence of a similar finding in Luminal B challenges the assumption of a uniform effect of prenatal hormones in all hormone receptor positive cancers. This raises questions about the complex mechanisms by which prenatal hormonal exposure may predispose to or protect against certain breast cancer phenotypes. This may reflect differences in biological structure and etiology between these subtypes that are not determined solely by hormone receptor status. For example, the proliferative index and the presence or absence of specific genetic markers may influence how prenatal hormonal exposure affects breast cancer risk. This underscores the complexity of breast cancer biology.

Breast cancer patients who are ER positive are more likely to be AR positive. Most studies have shown that AR expression in ER positive tumors is associated with better overall survival, longer relapse free survival, better response to endocrine therapies and lower Ki-67 positivity. These features are what we expect in Luminal A tumors within breast cancer subgroups. Furthermore, within the hormone receptor positive luminal subgroup, Luminal A tumors exhibited higher AR expression compared to Luminal B tumors. This differential AR expression between luminal subtypes may partially explain the different 2D:4D ratio findings in our study and suggests a deeper, more complex interaction between prenatal hormone exposure and tumor biology in Luminal A breast cancer.

This nuanced relationship between prenatal hormone exposure reflected in the 2D:4D ratio and differential AR expression in Luminal A and Luminal B subtypes provides a compelling avenue for further investigation. It suggests that prenatal hormonal exposure may predispose individuals to develop certain types of breast cancer, potentially through mechanisms that affect AR and ER expression patterns.

The higher AR expression status in Luminal A patients could be interpreted as a result of greater testosterone exposure during the intrauterine period in this patient group, leading to a lower 2D:4D ratio in the right hand. This explains our finding of a lower 2D:4D ratio in luminal A breast cancer patients.

In a number of rat experiments, it has been shown that receptor density in the fourth finger is higher than in the second finger. Testosterone has been shown to increase phalanx bone growth, while estrogen has been shown to decrease it. Since male foetuses secrete more testosterone, males have a lower 2D:4D ratio. It is accepted that the 2D:4D ratio reflects the balance between androgenic and estrogenic receptor stimulation or signalling rather than high or low testosterone levels^16^.

There are several lines of evidence suggesting that prenatal testosterone exposure or underlying sensitivity to testosterone is associated with 2D:4D, particularly right 2D:4D. Evidence from a mouse model suggests that the 2D:4D ratio is more dependent on androgen exposure than intrauterine estrogen exposure, with higher testosterone exposure relative to estrogen leading to lower 2D:4D in the right hand^16^. In a study by Udicki et al., a statistically significant higher 2D/4D ratio was observed only in the right hand in women with breast cancer compared to the healthy control group^6^. We also found that our study is consistent with many other reports showing that prenatal testosterone exposure or testosterone sensitization, especially right 2D:4D, is inversely associated^6,17,32–34^. Considering these studies, it may be considered that the ratio on the right hand may be a better indicator of breast cancer risk.

While our study provides valuable information, it is not without limitations. While the sample size is sufficient to detect differences in the Luminal A group, it may not have the power to distinguish associations in other subtypes. In addition, reliance on the 2D:4D ratio as an indirect measure of prenatal hormonal exposure, although widely accepted, does not account for the full spectrum of intrauterine hormonal effects. Furthermore, our study design fails to capture postnatal hormone fluctuations and their potential interaction with prenatal exposure in cancer development.

Despite these limitations, our study adds an important piece to the puzzle of understanding the etiologic heterogeneity of breast cancer. The significant association between the 2D:4D ratio and Luminal A subtype suggests that prenatal hormonal influences may have subtype-specific effects on breast cancer risk. Our finding opens new avenues for research into early life exposures and their long-term health effects.

Going forward, integrating the 2D:4D ratio with genetic, environmental and lifestyle factors may enrich breast cancer risk models, potentially leading to more personalized prevention strategies. Furthermore, our findings argue for a more nuanced approach to study hormone-dependent cancers, emphasizing the need to consider diversity within hormone receptor-positive subtypes.

In conclusion, our study sheds light on the potential link between prenatal hormone exposure and Luminal A breast cancer, while also highlighting the complexity of this disease and the need for more sophisticated research methodologies to fully understand its origins and determinants.

## Data Availability

The datasets analyzed in the current study are not publicly available due to the possibility to showcase patients identity. However, they could be available from the corresponding author upon reasonable request.
